# Plant-Based Diets and Metabolic Syndrome Components: The Questions That Still Need to Be Answered—A Narrative Review

**DOI:** 10.3390/nu16010165

**Published:** 2024-01-04

**Authors:** Klaudia Wiśniewska, Katarzyna Małgorzata Okręglicka, Aneta Nitsch-Osuch, Michał Oczkowski

**Affiliations:** 1 Department of Social Medicine and Public Health, Medical University of Warsaw, 3 Oczki Street, 02-007 Warsaw, Poland; katarzyna.okreglicka@wum.edu.pl (K.M.O.); aneta.nitsch-osuch@wum.edu.pl (A.N.-O.); 2 Doctoral School, Medical University of Warsaw, 02-091 Warsaw, Poland; 3 Department of Dietetics, Institute of Human Nutrition Sciences, Warsaw University of Life Sciences (SGGW-WULS), 159c Nowoursynowska Street, 02-776 Warsaw, Poland; michal_oczkowski@sggw.edu.pl

**Keywords:** metabolic syndrome, plant-based diet, vegan, vegetarian, obesity, weight loss

## Abstract

Metabolic syndrome (MetS) is defined as the co-occurrence of at least three of the following metabolic disorders: abdominal obesity, hypertriglyceridemia, low high-density lipoprotein cholesterol (HDL-C), high blood glucose, and hypertension. The treatment of MetS involves lifestyle changes, including following an appropriate diet. In addition to weight reduction, it is crucial to search for optimal nutritional patterns that are highly effective in optimizing other MetS markers, such as glucose and lipid metabolism, and reducing blood pressure. To date, the effects of a Mediterranean diet and Dietary Approaches to Stop Hypertension (DASH) diet on MetS have been extensively evaluated. Recent epidemiological studies suggest that plant-based diets (PBDs) may be effective in treating MetS; however, there is still a lack of experimental data. This review aims to analyze the potential benefits of different PBDs on MetS determinants based on the available studies. The findings may help personalize dietary interventions and improve patient care for those with MetS.

## 1. Introduction

Sedentary behaviors are associated not only with limited physical activity, but also with an increased consumption of high-energy and ultra-processed foods. These lifestyle changes contribute to a rise in the number of people suffering from obesity as well as a higher risk of hypertension and disorders of carbohydrate and lipid metabolism [1,2,3]. These disorders are associated with metabolic syndrome (MetS) which, in the absence of appropriate treatment, leads to the development of cardiovascular diseases and type 2 diabetes mellitus (T2DM) [4].

According to the International Diabetes Federation, the American Heart Association, and the National Heart, Lung, and Blood Institute [4], MetS can be defined as the presence of at least three of the following risk factors for the development of cardiovascular diseases and diabetes:

- Waist circumference exceeding 94 cm in men and 80 cm in women;
- High plasma triglycerides level—at least 150 mg/dL;
- Lowered plasma high-density lipoprotein cholesterol (HDL-C) level—less than 40 mg/dL in men or less than 50 mg/dL in women;
- Fasting plasma glucose level of at least l00 mg/dL;
- Systolic blood pressure (SBP) of at least 130 mmHg and/or diastolic blood pressure (DBP) of at least 85 mmHg.

The prevalence of MetS varies depending on the region and population being studied. The global MetS occurrence ranges from 12.5% (95% confidence interval (CI): 10.2–15.0) to 31.4% (29.8–33.0), with an upward trend being observed in most cases. Recent data published in 2022 [5] show a positive correlation between MetS prevalence and the country’s income level. An analysis of risk factors for MetS reveals a global prevalence at ca. 45% for ethnic-specific abdominal obesity and 24.5% for fasting plasma glucose ≥ 100 mg/dL. Additionally, a prevalence of 42.6% for SBP ≥ 130 mmHg and/or DBP ≥ 85 mmHg, 40.2% for HDL-C < 40 mg/dL was found for men, or <50 mg/dL for women, and 28.9% for serum triglycerides ≥ 150 mg/dL.

Prevalence data clearly indicate that MetS is a significant health issue with high healthcare costs. Early diagnosis and treatment are crucial [6,7,8]. One of the elements preventing MetS and supporting its treatment is a change in lifestyle, including following an appropriate diet [9]. In addition to weight reduction, it is crucial to search for optimal nutritional patterns that effectively optimize other components of MetS, such as glucose and lipid metabolism, and reduce blood pressure [9,10]. Regarding the recent recommendations [11], it seems reasonable to consider diets limiting the consumption of meat and meat products. Healthy plant-based diets (PBDs) have not only been associated with a lower risk of various non-communicable chronic diseases (NCDs) [12,13], but also seem to be more environmentally sustainable, as they may significantly reduce agricultural land use, greenhouse gas emissions, water usage and eutrophication [14].

To date, the effects of the Mediterranean diet and Dietary Approaches to Stop Hypertension (DASH) diet on MetS management have been extensively evaluated [15,16,17,18,19,20,21]. However, there have been very few analyses on the impact of PBDs, particularly from experimental studies. This paper focuses on the effects of typical PBDs, which exclude meat, fish, and seafood consumption (vegan, vegetarian, including ovo-vegetarian and lacto-ovo-vegetarian), and their effects on MetS, as presented in articles published in recent years. In addition, searches were performed for other, less restrictive PBDs, such as pescatarian, semi-vegetarian, and flexitarian diets. The aim of this review is to analyze the potential benefits of various PBDs on MetS components based on the available studies, since improving individual components may lead to better patient prognosis.

## 2. The Context: What Do We Know about Plant-Based Diets and Their Potential Effects on Metabolic Syndrome Components?

### 2.1. Plant Based Diet Characteristics

The term “plant-based diet” refers to a wide range of dietary patterns that involve the reduced consumption of animal-based foods, such as meat and dairy products, and increased consumption of plant-origin food items. The literature offers different interpretations of the various types of PBDs [22,23].

**Figure 1:**
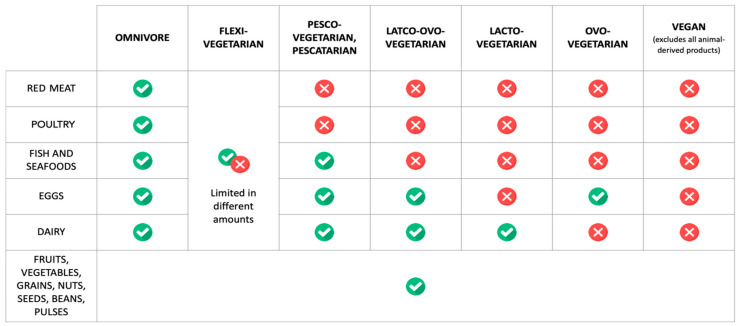
Classification of diets according to the amount of animal or plant products consumed.

Several authors suggest that PBDs do not require the exclusion of any food groups. That is why diets in which plant products constitute the dominant food source, such as the Mediterranean diet or the DASH diet, are often included in this group [24,25]. Other studies equate PBDs with vegetarianism. Therefore, it is crucial to adopt universal definitions of both these terms in order to limit the risk of faulty interpretations of the studies and incorrect conclusions. The same problem has been observed by Hargreaves et al. [26], who proposed defining PBD as “a dietary pattern in which foods of animal origin are totally or mostly excluded”. Such a definition includes those non-vegetarian diets in which calories from plant-based foods are dominant. A narrower definition was put forward for the vegetarian diet, which reads “a dietary pattern that excludes meat, meat-derived foods, and, to different extents, other animal products”. The basis of a vegetarian diet consists mainly of plant products, which include grain products, vegetables and fruits, legumes. In addition, nuts, seeds, and vegetable oils are consumed. Due to the potential negative impact on the MetS factors of processed, low-nutritional-value and high-salt plant food, low-processed food is highly recommended. On the other hand, recommendations for a higher consumption of vegetables to reduce the risk of MetS should also take into account the proper food processing methods (for example, freezing, blanching or steaming) that can help to diminish the loss of bioactive compounds (including polyphenols), which are crucial for the prophylaxis and dietary management of MetS components [27]. Vegetarian diets are categorized based on the type of products that are allowed or eliminated. These two definitions distinguish between vegetarian diets (ranging from lacto-ovo-vegetarian to vegan diets) and non-vegetarian diets that rely heavily on plant-based foods. Hence, the latter group includes flexitarian, pescetarian and ovo-lacto-vegetarian diets, providing that animal-based foods (meat, eggs or dairy products) in these diets do not constitute the primary food source and at least half of the energy value comes from plant products [22,26,28]. The PBDs that are the subject of this review, along with their definitions, are presented in Figure 1.

It should be added that the literature also discusses various subtypes of vegan diets, for instance, a raw vegan diet (no processed or cooked foods), whole-food vegan diet (no processed foods), and whole-food, low-fat vegan diet (no processed and high-fat plant foods) [26]. Some of them are analyzed in this review.

**Figure 2:**
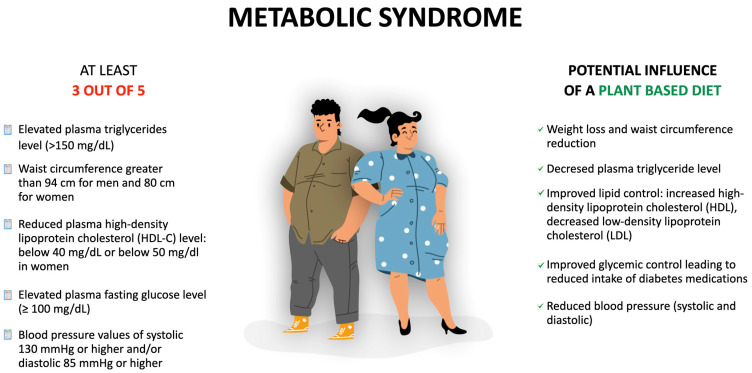
Plant-based diets and their potential influence on metabolic syndrome.

The American Dietetic Association [22] indicates that properly balanced and well-planned vegetarian/vegan diets are suitable for all stages of human life. Appropriately composed vegetarian diets provide all essential nutrients and help to prevent many non-communicable chronic diseases (NCDs). Furthermore, lacto-vegetarians and vegans have been shown to have higher overall diet quality compared to non-vegetarians [29,30]. Vegetarians are more likely to closely follow recommendations for total fruit, whole-grain, and plant-based protein consumption. Since vegetarian diets are characterized by an overall higher quality, this fact may, to some extent, account for the better health outcomes compared to omnivore diets [31]. Research shows that PBDs are often linked with improvements in many parameters, including MetS markers or factors caused by them [32,33,34]. The potential impact of plant-based diets on metabolic syndrome is presented in Figure 2.

### 2.2. PBDs Potential Effects on the Components of MetS

Different types of PBDs may have varying effects on MetS parameters. Since research comparing all MetS parameters is scarce, the present paper discusses studies focused primarily on MetS components.

#### 2.2.1. Waist Circumference and Body Mass

It has already been established that the implementation of vegetarian/vegan diets resulted in a reduction in mean body weight among the participants in clinical trials, which points to their usefulness in preventing and managing weight-related conditions [35]. As weight decreases, so does waist circumference, but not all studies report these parameters. Interesting results were provided by a study conducted by Turner-McGrievy et al. [36], in which obese and middle-aged subjects were randomly assigned, for the duration of 6 months, to one of the following diets: omnivorous, semi-vegetarian, pesco-vegetarian, vegetarian, or vegan, without any restrictions regarding food quantity. After this period, the greatest body weight reduction was noticed in the vegan (−7.5%) and the vegetarian (−6.3%) subjects compared to the other groups (about −3.2%). This was the first study of this type comparing the effectiveness of different PBDs. A similar trend was also observed in other studies [37,38,39,40,41]. Further publications, including meta-analyses, concentrated mostly on vegan and vegetarian diets. A meta-analysis of randomized controlled trials by Huang et al. [42] found that following vegetarian diets may be conducive to weight loss. A significant weight reduction was observed in patients following a vegan diet (−2.52 kg; 95% CI: from −3.02 to −1.98), while those following lactovegetarian diets experienced a lower weight reduction (−1.48 kg; 95% CI: from −3.43 to 0.47). Greater weight loss was noted among patients on calorie-restricted vegetarian diets (−2.21 kg; 95% CI: from −3.31 to −1.12) compared to those without energy restriction (−1.66 kg; 95% CI: from −2.85 to −0.48). Increased weight loss was also observed in those individuals with a follow-up period of <1 year (−2.05 kg; 95% CI: from −2.85 to −1.25) compared to those with a follow-up period of ≥1 year (−1.13 kg; 95% CI: from −2.04 to −0.21). A recent meta-analysis confirmed a positive relationship between following PBDs and body composition [43,44,45,46]. However, some of the analyzed studies showed no significant differences in changes in patients’ body weight, BMI, and waist circumference, especially when vegan and vegetarian diets were compared with other beneficial dietary patterns, such as the Mediterranean diet [47] or the recommendations of the American Heart Association [43]. Melgar et al. [44] also observed a reduction in body weight (by 3.60 kg, 95% CI from −4.75 to −2.46), without a significant reduction in waist circumference, in individuals who reported a vegetarian dietary pattern. The results of clinical trials, as well as meta-analyses of these studies, are, therefore, inconsistent. The authors agree that vegetarian diets, including vegan diets, can lead to weight loss, but the results on body composition or waist circumferences are inconclusive. The potential benefits of PBDs for body weight and other anthropometric parameters may result from a better diet structure, such as a low caloric density and reduced levels of saturated fatty acids, as well as a higher content of fiber and mono- and polyunsaturated fats [45]. Other, not fully explained mechanisms may concern the role of intestinal microbiota, the obesity-causing effects of trimethylamine *n*-oxide, or the role of polyphenols on the regulation of fatty acid metabolism [46].

#### 2.2.2. Lipid Metabolism

PBDs have also been associated with positive cardio-metabolic effects. In fact, PBDs lead to a reduced risk of cardiovascular disease, cardiovascular disease mortality, and all-cause mortality [17], though most of the data come from large prospective cohort studies carried out in the USA, Europe, or Asia [48], not from experimental studies. However, even for cohort studies, these correlations are not observed for every population. For instance, in a study of black Americans, no positive correlation was found between higher adherence to a PBD and a decrease in mortality from cardiovascular causes or any cause [13]. Important results were also obtained by Termannsen et al. [49], showing a significant reduction in total cholesterol (−0.30 mmol/L, 95% CI: from −0.52 to −0.08, *p* = 0.007), and low-density lipoprotein cholesterol (LDL-C) (−0.24 mmol/L, 95% CI: from −0.40 to −0.07). However, the effect was not observed for HDL-C, triglycerides [50]. On the other hand, a meta-analysis of studies from 1982 to 2022, published in 2023 [51], strongly indicated that a vegan diet, compared to omnivorous diets, was associated with reduced TC, LDL-C levels. These findings were consistent with results obtained from RCT studies reviews published through 2017 [52]. The cardioprotective effect of PBDs is primarily due to the low levels of saturated fatty acids, nitrosamines, and heme iron, commonly found in meat products. In addition, the higher intake of dietary fiber in these diets has the effect of lowering plasma cholesterol levels by binding dietary cholesterol to bile acids in the intestinal. Subsequently, the decrease in cholesterol level results in increased LDL receptor expression and activity and the removal of LDL from plasma. Another important factor is an increased intake of polyphenols, including flavonoids commonly found in fruits, vegetables, and legumes. There is a wide range of evidence regarding the involvement of these compounds in reducing lipid levels, including their ability to increase resistance to LDL oxidation and inhibit thrombus formation [45,46,48,53].

#### 2.2.3. Blood Pressure

Inappropriate dietary habits have been linked to an increased risk of hypertension [54]. Moreover, excessive salt intake is a well-documented dietary factor that significantly increases the risk of this disease [55]. However, the results of studies on the effects of specific types of plant-based diets on hypertension are inconsistent. In the previously mentioned study by Weston et al. [13], no decrease in blood pressure values was observed. With regards to hypertension, Gibbs et al. [56] showed that PBDs with limited animal products lead to a reduction in both SBP and DBP values, irrespective of the participant’s gender and BMI. The pooled analysis revealed that PBDs in vegan and lacto-ovo-vegetarian subjects were associated with a lower SBP (by 1.30 mmHg, 95% CI: –3.90, 1.29; and 5.47 mmHg, 95% CI: –7.60–3.34, respectively). Similar effects were noted for DBP. Dybvik et al. [57] indicated that vegetarians and vegans had a lower risk (by 15% and 18%, respectively) of hypertension compared to omnivores. The beneficial effects of PBDs on blood pressure also result from a higher intake of hypotensive dietary components, such as dietary fiber, potassium, magnesium, and calcium. Furthermore, compared to omnivores, individuals following a plant-based diet tend to consume less salt. Salt restriction is an effective way to prevent and treat hypertension [57,58].

#### 2.2.4. Glycemia

Reducing the consumption of meat and animal origin products has been linked to a decreased risk of certain NCDs, such as T2DM. Termannsen et al. [49] demonstrated that vegan diets led to a reduction in plasma glycated hemoglobin (HbA1c) level (−0.18%, 95% CI: from −0.29 to −0.07) compared to control diets. Different results were provided by the study by Melgar et al. [44], indicating that a vegan diet was not associated with a decrease in the plasma HbA1c or insulin level. One of the main mechanisms responsible for an improved glycemic control in people following PBDs is the improvement in insulin sensitivity. This beneficial effect can be brought about by the consumption of foods with a lower glycemic index, such as legumes and green leafy vegetables, as well as the replacement of meat with legumes, including soy products [59]. Glycemic control may also be improved by the gastrointestinal hormonal response, particularly the incretins response. These hormones, such as glucose-dependent insulinotropic peptide, peptide tyrosine-tyrosine and pancreatic peptide, are involved in the regulation of glucose metabolism and satiety. In patients with T2DM, the action of incretins is impaired, which may be further influenced by diet. Belinova et al. [60] demonstrated that the consumption of processed meat limits the release of gastrointestinal hormones, including incretins, both fasting and after a meal, compared to a vegan meal. A similar effect was reported by Klementova et al. [61], following a randomized crossover study that showed an increase in plasma gastrointestinal hormones and greater satiety after eating a single plant-based meal containing tofu compared to a meal consisting of processed meat and cheese with adequate energy and macronutrient content. These findings suggest that PBDs may be beneficial for improving gastrointestinal hormone release in diabetic patients. While plant-based diets have many benefits, it is important to consider the degree of processing of plant products, as well as the sugar content and total dietary sugar intake. Plant-based diets, particularly vegan diets, tend to have a higher carbohydrate intake, which may result in a higher proportion of sugar in the diet. This can have a negative impact on individual MetS components, including glycemia [3,15,16]. In particular, the findings demonstrated by Åbergg et al. [62] revealed that the intake of less-processed whole-grain products with a lower glycemic index (compared to finely milled whole-grain foods) contributed to a lower variability in 24 h glycemia in patients with T2DM.

## 3. Plant-Based Diets and Their Influence on Metabolic Syndrome Indices—Current Evidence from RCTs Studies

This review presents the current evidence on the outcomes of PBD treatments on MetS components to determine which supportive strategies and dietary treatments are effective and therefore may be recommended for implementation in clinical practice. A summary of the results of RCTs studies is presented in Table S1 (in the Supplementary Material). It should be noted that this does not include data on meat-restrictive diets such as the DASH diet and the Mediterranean diet, which have been extensively reported elsewhere [15,17,19,21,25].

Additionally, there data from randomized clinical trials concerning the effects of vegetarian diets (lacto-vegetarian, lacto-ovo-vegetarian, ovo-vegetarian) on MetS parameters are scarce. Thomas et al. [63] conducted a randomized crossover study among people following PBDs. The study recruited participants diagnosed with MetS. They were assigned a vegetarian diet for 13 weeks with 2 eggs per day, or an equivalent amount of egg substitute combined with spinach in the form of an omelet for breakfast. All participants followed a lacto-vegetarian diet ad libitum, restricting meat, poultry, and fish. In addition, eligible participants underwent a 2-week wash-out period without eggs and spinach intake. They were randomly assigned to eat spinach (70 g) with 2 eggs or an equivalent amount of egg substitute for breakfast for 4 weeks. After a 3-week washout period, they were assigned an alternative breakfast. As intended by the researchers, spinach was provided as a good source of lutein, zeaxanthin, and choline, and was also offered with whole eggs or egg substitute to assess whether the fats in the egg yolk would further promote the absorption of these nutrients. Decreased body weight (*p* < 0.02) and a higher HDL-C (*p* < 0.025) were observed as a result of the egg-inclusive diet. There were no differences in plasma glucose, insulin, LDL-C, triglycerides level, and blood pressure value. The plasma HDL-C level was higher in the ‘egg-inclusive diet’ patients (*p* < 0.01) compared to ‘the egg-substitute’ ones. These results indicate that a combination of a PBD and whole eggs may increase plasma HDL-C levels as well as plasma choline and zeaxanthin levels, important biomarkers in people with MetS. In the CARDIVEG study [47], no significant difference was noticed in weight reduction between the patients following the lacto-vegetarian diet and the Mediterranean diet. However, differences were observed in biochemical parameters. The lacto-vegetarian diet led to a greater decrease in plasma LDL-C level (−5.44%) compared to the Mediterranean diet. In contrast, the latter diet showed a significant decrease (−5.91%) in triglyceride levels.

The study by Mishra et al. [64] concerned the effectiveness of PBDs in obesity treatment and metabolic disturbances management. It demonstrated that dietary intervention resulted in significant improvements in body weight, plasma lipid profile and glycemic control among diabetics. The changes in these variables in the intervention group were higher than in the control group, and were statistically and clinically significant. Moreover, in the majority of cases, the differences between groups remained significant after considering gender, baseline values, cluster, and medication changes. Although many participants in the intervention group were less than fully adherent to the recommended diet, the alterations introduced in their diet were still significant, and changes in anthropometric and clinical variables were observed.

Different findings were reported by Lee et al. [65], who showed that statistical significance was only achieved for the plasma HbA1c level. A significant reduction (by −0.5% and −0.2%) in the HbA1c level was detected in the group of vegan and omnivores, respectively. In addition, the vegan diet appeared to be more effective in terms of glycemic control among T2DM patients. No statistically significant differences were observed for anthropometric parameters (BMI, waist circumference). Both groups displayed similar changes in LDL-C and HDL-C, as well as SBP and DBP, while triglyceride levels tended to increase in the vegan diet group and to decrease in the conventional diet group.

A lack of significant changes between groups in lipids and anthropometric parameters was also recorded by Kahleova et al. [40]. Significant reductions in total LDL, HDL-C and BMI were only observed in the intervention group, which also showed a decrease in basal insulin secretion. Furthermore, the patients from the intervention group exhibited slightly increased beta-cell sensitivity to glucose, although statistical significance was not achieved. Furthermore, no significant changes in beta-cell function parameters were noted in the control group, with the exception of an increase in total insulin secretion. The intervention group also showed a significant dose-dependent increase in insulin secretion as a function of plasma glucose concentration.

Jenkins et al. [50] compared metabolic parameters in subjects following a low-carbohydrate vegan versus a high-carbohydrate lacto-ovo-vegetarian diet following a 6-month observation. The treatment was preceded by a 1-month-long metabolic version of these diets. It was observed that weight loss of approx. 4 kg following the metabolic study was increased to −6.9 kg on low-carbohydrate and −5.8 kg on high-carbohydrate diets after 6-month ad libitum treatments (treatment difference 95% CI: −1.1 kg, *p* = 0.047). The relative LDL-C and triglyceride plasma level reductions were also greater when following the low-carbohydrate treatment (treatment difference 95% CI: −0.49 mmol/L, *p* < 0.001 and −0.34 mmol/L, *p* = 0.005). No significant effect on other metabolic outcomes was noted. The authors concluded that a self-selected low-carbohydrate vegan diet was more conducive to a decrease in lipid levels than a high-carbohydrate, low-fat weight loss diet, and therefore had a greater capacity to mitigate heart disease risk factors. It should be also pointed out that the study had a small group of participants (*n* = 39) and a high dropout rate. Hence, further trials are warranted to evaluate the effect of low-carbohydrate diets, including more plant-based low-carbohydrate diets, on metabolic parameters.

Wright et al. [66] examined the effects of introducing dietary interventions regarding a whole-food plant-based (WFPB) diet, comparing it with standard dietary treatment in New Zealand (control group). After 6 months of the study, the intervention group showed a mean reduction in BMI of 4.4 kg/m^2^ (range 0.4–7.4, 95% CI 3.7–5.1) and a mean reduction in body weight of 12.1 kg (range 1.4–27.3, 95% CI 9.9–14.3). Within the control group, there were no significant BMI reductions after 3 or 6 months of observation. At 6 months, the between-group analyses showed differences in BMI of 3.9 kg/m^2^ (95% CI ± 1), and in body weight of 10.6 kg (95% CI ± 2.9), which favored the intervention. The intervention group also showed a statistically significant mean reduction in total cholesterol at all time periods, although there was a smaller effect size observed with time: at month 3, it was 0.95 mmol/L (95% CI: 0.51–1.39, *p* < 0.001), while at month 6, it was 0.71 mmol/L (95% CI: from 0.28 to 1.14, *p* < 0.01). Comparing standard care and the interventional diet program at 6 months, the analysis showed a non-significant reduction in total cholesterol at 0.45 (95% CI: from −0.09 to 1.00). Differences in plasma HbA1c level between patient groups were more favorable for the intervention group, with a reduction of 5 mmol/L (95% CI: ±3, *p* < 0.001) at 6 months. In the intervention group, waist circumference decreased from baseline at all time periods. No change was observed in the control group, and differences between groups showed a 10 cm (95% CI: ±4, *p* < 0.0001) higher mean reduction in intervention at 6 months. After 6 months, the mean BMI reduction was higher with the WFPB diet compared to normal care (4.4 vs. 0.4, difference: 3.9 kg m^−2^ (95% CI: ±1, *p* < 0.0001).

A study by Crosby et al. [67] showed statistically significant improvements in anthropometric and biochemical parameters exhibited by the low-fat vegan intervention group compared to the control. It should be noted that participants in the control group were not required to follow any dietary recommendations and did not receive standard medical care. While the parameters in the control group remained stable, the following results were reported for the vegan group: a decrease in body weight (5.9 kg; 95% CI: from 6.8 to 5.0) and fat mass (4.1 kg; 95% CI: from 4.7 to 3.5), as well as a reduction in visceral fat volume. Insulin resistance parameters also decreased as a result of the low-fat vegan diet (treatment effect: 1.2; 95% CI: 2.2–0.3; *p* < 0.008). In addition, an improvement in insulin sensitivity was observed. This study showed significant changes in energy and nutrient intake, which had a positive impact on diet quality.

In contrast, a study conducted on individuals with ischemic heart disease found no significant changes after following a vegan diet in biochemical and anthropometric parameters compared to the standard American Heart Association (AHA) recommendations [43]. Decreases in body weight, as measured by BMI and waist circumference, were observed in both the vegan diet and in participants following the AHA recommendations. However, these changes were not statistically significant between the two groups. In addition, there were no significant differences in fasting glucose, HbA1c, insulin and blood lipids between the groups. The authors concluded that a vegan diet does not provide significant additional benefits over the AHA-recommended diet in terms of weight loss, glycemic control, or improved lipid profile. Still, a vegan diet may be recommended to reduce inflammation, as measured by the high-sensitivity C-reactive protein, a key factor in the development of major adverse cardiovascular events.

To the best of our knowledge, there are no current RCTs analyzing flexitarian and semi-vegetarian diets on MetS-related metabolic parameters. Most of the data came from epidemiological studies [68].

## 4. Future Perspective

The present review identified several issues with the clinical studies discussed in terms of their suitability for evaluating the effects of PBDs on MetS. First, only a limited number of the analyzed studies considered all parameters of MetS, while most of them focused only on selected MetS markers [64,65]. Another major problem is the heterogeneity of the studies in question, with variability already being present in the control groups. In some cases, the control groups followed country-specific dietary recommendations [40,65,66], while in other studies no nutritional guidelines were provided [40,64,67]. A better approach would be for the control group to follow an optimal dietary model developed for the country or at least to adhere to general world recommendations [69]. Moreover, the intervention groups consisted of diverse participants with varying health statuses (healthy people and persons with T2DM), which renders the task of comparing the effectiveness of different interventions even more challenging. As the interventions themselves ranged from simple to more complex, the issue of heterogeneity also pertains to delivery mechanisms, intensity, and behavioral strategies. Variations were also recorded in the supplementation protocol or the lack thereof. Following a vegetarian and especially a vegan diet may result in deficiencies in micronutrients. One example of a deficient vitamin that should be supplemented, especially in the vegan group, is vitamin B12 [48,70,71]. Some studies lacked supplementation data and information on what doses and forms of vitamin B12 were provided to participants [43,50,52].

Finally, when designing experimental clinical trials, it is important to consider the differences in the amount of animal- and plant-origin products. Hargreaves et al. [26] pointed out that although vegetarians tend to consume more plant foods, a vegetarian diet may nonetheless contain a significant number of calories from non-plant-based sources (dairy, eggs, honey). The percentage of calories from animal products may be even higher in the case of flexitarian, pescetarian, and semi-vegetarian diets. This is especially critical when designing studies in which patients consume foods ad libitum.

Therefore, it is impossible to conclude, based on RCT studies, which PBD intervention is most effective in patients with MetS. The considerable heterogeneity of the reviewed studies and the limited comparisons between trials greatly limit the potential to implement the results in clinical practice. The studies were short- and medium-term, and the results should be verified in larger, long-term studies using consistent outcome measures. Future studies should also include a thorough description of the study protocol with information on dietary supplementation, an accurate assessment of the intake or diet that patients followed, and patient-related outcomes. It would also be beneficial to include an assessment of the patient’s quality of life during the intervention. In addition, future studies should aim to recruit larger groups of participants that are more diverse in terms of their race or ethnicity, socio-economic characteristics, and degree of adherence to a healthy lifestyle. Data collected in this manner could help to personalize nutritional interventions and improve the effectiveness of care for MetS patients.

## 5. Conclusions

The benefits of PBDs are significant both in terms of maintaining proper health and environmental protection. Well-planned PBDs are characterized by a higher proportion of health-promoting components, such as dietary fiber, mono- and polyunsaturated fatty acids, polyphenols, vitamins, mineral elements, and plant protein.

The findings analyzed in the present review indicate that PBDs are associated with beneficial effects on MetS components. Such diets, especially vegan and lacto-vegetarian ones, are associated with a decrease in body weight and waist circumference, an optimization of lipid parameters, a decrease in plasma glucose level and a lowered blood pressure.

However, there is a lack of high-quality RCTs to determine the best evidence-based dietary model of PBDs that can be recommended for the treatment of MetS. This review additionally identified important differences in study protocols that may affect the results. Therefore, further dietary intervention studies, optimally long-term ones, are necessary to evaluate the beneficial effects of plant-based diets to improve the care of patients with MetS.

## Data Availability

No new data were created or analyzed in this study. Data sharing is not applicable to this article.

## Supplementary Materials

The following supporting information can be downloaded at: https://www.mdpi.com/article/10.3390/nu16010165/s1, Table S1: The summary of the results of chosen RCT studies of different types of plant-based diets on parameters of the metabolic syndrome.

## Abbreviations

AHA	American Heart Association
BMI	Body Mass Index
CI	Confidence interval
DASH	Dietary Approaches to Stop Hypertension
DBP	Diastolic blood pressure
HbA1c	Glycated hemoglobin
HDL-C	High-density lipoprotein
LDL-C	Low-density lipoprotein
MetS	Metabolic Syndrome
NCDs	Non-communicable chronic diseases
PBDs	Plant-based diets
RCTs	Randomized controlled trials
SBP	Systolic blood pressure
T2DM	Type 2 diabetes mellitus
WFPB	Whole-food plant-based diet
