# Supplementary material for: Plant-Based Diets and Metabolic Syndrome Components: The Questions That Still Need to Be Answered—A Narrative Review
Source: Nutrients. 2024 Jan 4;16(1):165. doi: 10.3390/nu16010165 (PMC10780839; doi:10.3390/nu16010165)
Supplement: Supplementary file 1 [file nutrients-16-00165-s001.zip › nutrients-2748562-supplementary.pdf]

Supplementary materials

Plant-Based Diets and Metabolic Syndrome Components: The Questions That Still Need to Be Answered—A Narrative Review

Klaudia Wiśniewska [1,2,*], Katarzyna Małgorzata Okręglicka [1], Aneta Nitsch-Osuch [1] and Michał Oczkowski [3]

Table S1. The summary of the results of chosen RCT studies of different types of plant-based diets on parameters of the metabolic syndrome

Author Year, (reference)	Sample Size	Study Duration (weeks)	Age (years)	BMI (kg/m2)	Intervention and Comparator (control) Treatments	Food intake information (calorie restriction or ad libitum)	Main Conclusions
Mishra, 2013 [64]	VD – 142<br>C – OM	18	45.2 (14.5)	35.0 (0.7)	**Intervention:** low fat vegan diets<br>**Comparator:** no change in dietary patterns	Ad libitum	The low fat vegan diet led to significant improvements in body weight, plasma lipids and glycemic control among diabetics.
Jenkins, 2014 [50]	V - 20<br>C - 19	24	NA	31.1	**Intervention:** low-carbohydrate vegan diet containing 26% of calories from carbohydrate, 31% of calories from vegetable proteins and 43% from fat<br>**Comparator:** high-carbohydrate lacto-ovo-vegetarian diet (58% carbohydrate, 16% protein and 25% fat)	Ad libitum, but participants were encouraged to eat only 60% of their estimated caloric requirements	Compared to a high-carbohydrate diet, a self-selected low-carbohydrate vegan diet was more beneficial in lowering lipids and, by extension, improving heart disease risk factors.
Lee, 2016 [65]	V - 46<br>C - 47	12	57.9 (7.3)	23.5 (3.0)	**Intervention:** vegan diets<br>**Comparator:** conventional diet recommended by the Korean Diabetes Association 2011	Ad libitum	Both diets led to a reduction in HbA1c levels, but the vegan diet was more effective in glycemic control among patients with T2DM compared to the conventional diabetic diet recommended by the Korean Diabetes Association.
Wright, 2017 [6]	V – 33<br>C - 32	24	**Intervention**: 56 (9.9)<br>**Comparator**: 56 (9.5)	**Intervention**: 29<br>**Comparator**: 30	**Intervention:** low-fat vegan diet (approximately 7–15% total energy from fat)<br>**Comparator:** standard medical care in New Zealand	Ad libitum	A low-fat vegan diet led to significant improvements in BMI, WC, cholesterol levels and other cardiovascular risk factors compared to the standard medical care.
Kahleova, 2018 [40]	V – 38<br>C – 37	16	**Intervention**: 52.6 (14.7)<br>**Comparator**: 54.3 (9.9)	**Intervention:** 33.1 (31.8–34.3)<br>**Comparator**: 33.6 (32.5–34.8)	**Intervention:** low-fat vegan diet (~75% of energy from carbohydrates, 15% protein, and 10% fat)<br>**Comparator:** no change in dietary patterns	Ad libitum	A low-fat vegan diet affects significant changes in HOMA-IR, body weight and BMI compared to the control group. Beta-cell function and insulin resistance were modified by the dietary intervention.
Sofi F, 2018 [47]	VD – 60<br>C - 58	12	51.1	30.6 (4.9)	**Intervention:** lacto-ovo-vegetarian diet (~50% to 55% of energy from carbohydrate, 25% to 30% from total fat, 15% to 20% from protein)	Calorie restriction	Both VD and MD were significantly effective in reducing BW, BMI and fat mass, but with no significant differences between them. The lacto-vegetarian diet was more effective in reducing low-density lipoprotein cholesterol levels. On

					**Comparator:** MD (~50% to 55% of energy from carbohydrate, 25% to 30% from total fat, 15% to 20% from protein)		the other hand, MD led to a higher reduction in triglyceride levels.
Shah, 2018 [43]	V – 50<br>C – 50	8	**Intervention**: 63.0<br>**Comparator**: 59.5	**Intervention**: 30.5<br>**Comparator**: 29.4	**Intervention:** vegan diet<br>**Comparator:** conventional diet recommended by the American Heart Association	Ad libitum	The efficacy of the vegan diet was not higher compared to the standard AHE recommendations. Differences in BMI, WC and other lipid parameters were not significant between the two diet groups. In patients with ischaemic heart disease, a vegan diet may be considered to reduce C-reactive protein levels with high sensitivity as a marker of adverse effect risk.
Crosby, 2022 [67]	V – 117<br>OM – 102	16	**Intervention**: 52.6 ± 12.8<br>**Comparator**: 56.5 ± 9.7	**Intervention**: 33.3 (32.6-34.0)<br>**Comparator**: 33.7 (32.9-34.4)	**Intervention:** low-fat vegan diet (~10% of energy from fat)<br>**Comparator:** no change in dietary patterns	Ad libitum	Increased intake of low-fat plant foods and decreased intake of high-fat and animal foods in the intervention group was associated with decreased BW and fat loss. A low-fat vegan diet may improve indicators of diet quality and metabolic health.
Thomas, 2022 [63]	24	13	49.3 ± 8	34.3 ± 4.6	**Intervention:** lacto-vegetarian diet including 2 eggs per day with spinach<br>**Comparator:** lacto-vegetarian diet with the equivalent amount of 2 eggs per day substitute in combination with spinach as an omelet for breakfast	Ad libitum	A lacto-ovo-vegetarian diet with whole eggs compared to vegan and lacto-vegetarian diets in participants with MetS appears to maintain and/or improve dyslipidaemia and markers of oxidative stress and inflammation.

Abbreviations: V, vegan; VD, vegetarian; OM, omnivorous; MD, Mediterranean diet; NA, not available; BMI, body mass index; BW, body weight; WC, waist circumference; HOMA-IR, Homeostatic Model Assessment of Insulin Resistance; HbA1c, glycated hemoglobin, T2DM, type 2 Diabetes Mellitus.
